# A Neutralizing Anti-gH/gL Monoclonal Antibody Is Protective in the Guinea Pig Model of Congenital CMV Infection

**DOI:** 10.1371/journal.ppat.1004060

**Published:** 2014-04-10

**Authors:** Marcy R. Auerbach, Donghong Yan, Rajesh Vij, Jo-Anne Hongo, Gerald Nakamura, Jean-Michel Vernes, Y. Gloria Meng, Samantha Lein, Pamela Chan, Jed Ross, Richard Carano, Rong Deng, Nicholas Lewin-Koh, Min Xu, Becket Feierbach

**Affiliations:** 1 Department of Infectious Diseases, Genentech, South San Francisco, California, United States of America; 2 Department of Translational Immunology, Genentech, South San Francisco, California, United States of America; 3 Department of Antibody Engineering, Genentech, South San Francisco, California, United States of America; 4 Department of Biochemical and Cellular Pharmacology, Genentech, South San Francisco, California, United States of America; 5 Department of Biomedical Imaging, Genentech, South San Francisco, California, United States of America; 6 Department of Clinical Pharmacology, Genentech, South San Francisco, California, United States of America; 7 Department of Biostatistics, Genentech, South San Francisco, California, United States of America; University of California, Davis, United States of America

## Abstract

Human cytomegalovirus (HCMV) is the most common cause of congenital virus infection. Congenital HCMV infection occurs in 0.2–1% of all births, and causes birth defects and developmental abnormalities, including sensorineural hearing loss and developmental delay. Several key studies have established the guinea pig as a tractable model for the study of congenital HCMV infection and have shown that polyclonal antibodies can be protective [1]–[3]. In this study, we demonstrate that an anti-guinea pig CMV (GPCMV) glycoprotein H/glycoprotein L neutralizing monoclonal antibody protects against fetal infection and loss in the guinea pig. Furthermore, we have delineated the kinetics of GPCMV congenital infection, from maternal infection (salivary glands, seroconversion, placenta) to fetal infection (fetus and amniotic fluid). Our studies support the hypothesis that a neutralizing monoclonal antibody targeting an envelope GPCMV glycoprotein can protect the fetus from infection and may shed light on the therapeutic intervention of HCMV congenital infection in humans.

## Author Summary

Human cytomegalovirus (HCMV) is the most common cause of congenital virus infection and causes developmental abnormalities, including hearing loss and developmental delay. Although there is no therapy for congenital HCMV disease, there is evidence from both human and animal studies that antibodies can have efficacy in this setting. Such studies have focused exclusively on polyclonal antibodies, in which the targets of protective antibodies are unknown. Guinea pigs have been used as a model of human maternal fetal transmission of infection because of similarities in placental anatomy between human and guinea pig. Furthermore, guinea pig CMV (GPCMV) has been demonstrated to cross the placenta and cause fetal infection and loss, similar to the effects of infection with HCMV. However, the kinetics of maternal and fetal infection in this model has not been carefully investigated. In this work, we have delineated the kinetics of maternal to fetal infection and found that congenital infection is rapid following maternal infection. Importantly, we demonstrate that a monoclonal antibody against a protein critical for viral entry protects pregnant guinea pigs against fetal infection. Thus, our studies may be informative for development of a therapeutic intervention to treat congenital HCMV infection in humans.

## Introduction

Human cytomegalovirus (HCMV), a member of the herpesvirus family, is widely distributed in the human population and can cause severe disease in immunocompromised patients and upon infection of the fetus. A therapeutic for HCMV infection is a major public health priority for women with primary HCMV infections during pregnancy. Congenitally-infected infants have a high incidence of neurodevelopmental sequelae, including mental retardation and sensorineural deafness [4]–[7]. Several lines of evidence suggest that neutralizing antibodies can protect the fetus from HCMV infection and disease. First, preconception maternal antibodies to HCMV significantly reduce the severity and risk of congenital HCMV infection in future pregnancies, although the frequency of sensorineural deafness is the same [6], [8]. Second, early appearance of maternal antibodies against the HCMV glycoprotein entry complex, gH/gL/UL128/UL130/UL131, correlates with a lack of vertical transmission, suggesting that antibodies against this complex can effectively neutralize the virus in vivo [9], [10]. And third, a small, open-label study showed that hyperimmuneglobulin could protect the fetus from HCMV infection and disease [11]. However, hyperimmuneglobulin is not an optimal therapy because of lot-to-lot variability, the possibility of inadvertent transmission of infection, the volumes that must be administered, and difficulties in maintaining an adequate supply. A monoclonal antibody with therapeutic efficacy would overcome all these problems, but there is no proof of concept that antibody against a single CMV epitope could confer protection against fetal infection. Therefore, we set out to test this hypothesis.

The guinea pig has been a useful model for the study of maternal-fetal transmission because the placental anatomy is similar to that of humans [12]–[15]. However HCMV does not infect guinea pigs, thereby necessitating the use of guinea pig CMV (GPCMV). GPCMV has been demonstrated to cross the placenta and cause fetal infection. Several studies have delineated a role for antibodies in the prevention of GPCMV infection and disease in the guinea pig [1]–[3] In early studies, Bia et al. demonstrated that preconception infection protected against in utero GPCMV transmission, most likely due to the generation of neutralizing antibodies in the mother [16], [17]. More recently, Bourne et al., determined that preconception immunization of pregnant guinea pigs with GPCMV glycoproteins protected the fetus from death and infection [2]. In addition, two studies determined that passive immunization of anti-GPCMV antibodies could be protective in the congenital setting [1], [3].

In this study, we determine whether a monoclonal antibody can confer protection in the congenital infection setting. To this end, we generated neutralizing monoclonal antibodies against GPCMV. We show that a neutralizing monoclonal antibody against the GPCMV gH/gL glycoprotein entry complex, when administered prophylactically to mothers with primary infection, protects against fetal infection and death. In addition, we characterize the kinetics of congenital infection, from maternal infection (seroconversion, salivary glands and placental infection) to fetal infection (fetus and amniotic fluid) and find that congenital infection is rapid following infection of the mother. These studies support the hypothesis that a neutralizing antibody response that targets a single epitope on a glycoprotein entry complex can be protective in the context of congenital infection.

## Results

### Kinetics of seroconversion in pregnant guinea pigs

**Figure 1:**
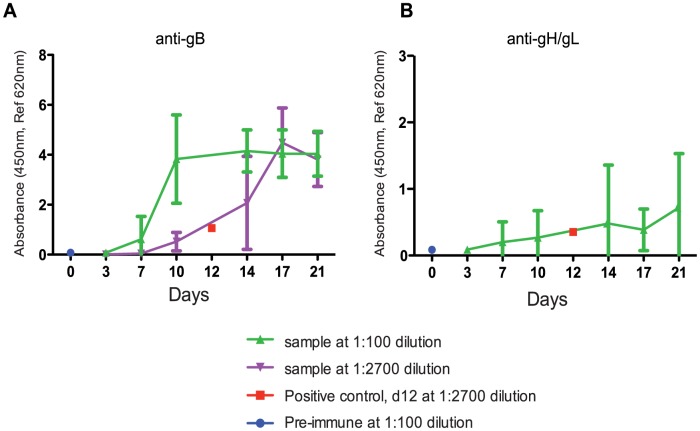
Determination of the timing of seroconversion. An antigen-based ELISA assay was used to monitor the anti-gB (A) or anti-gH/gL (B) IgG response in infected pregnant guinea pigs over 21 days. Two dilutions (1∶100- green, 1∶2700- purple) are graphed to display the range of reactivity of the anti-gB response. The positive control, day 12 positive serum, (1∶2700- red, average value from 5 different plates with 2 replicates per plate) consistently resulted in the linear range and near the middle of the dynamic range. Pre-immune samples were consistently at zero indicating the cut-off of the assay (an example is shown at d0 at 1∶100, blue). The 1∶2700 was not graphed for anti-gH/gL (B) due to lack of signal. The error bars represent standard deviation of the means calculated from the results of several animals at each time point (see methods for study details).

To understand the timing of seroconversion during pregnancy, GPCMV-free pregnant guinea pigs were inoculated with 4×10^3^ PFUs of pathogenic salivary gland-passaged GPCMV (*i*n *v*ivo *p*assage 8, IVP8) at day 21 of gestation (beginning of the second trimester). 4×10^3^ PFUs was empirically determined to result in robust fetal infection with minimal dam mortality (data not shown). Antibodies against GPCMV soluble gB or gH/gL protein could be detected by day 7, indicating primary infection, and continued to rise throughout the study (Figure 1). Reactivity against gB protein in serum samples tested at 1∶100 dilution appeared to plateau at day 10. However, additional analysis of samples diluted to 1∶2700 revealed that reactivity against gB protein continued to increase beyond day 10 (Figure 1A). Though minimal, serum reactivity against soluble gH/gL antigen gradually increased over the course of the study as well (Figure 1B).

### Kinetics of GPCMV infection in pregnant guinea pigs

**Figure 2:**
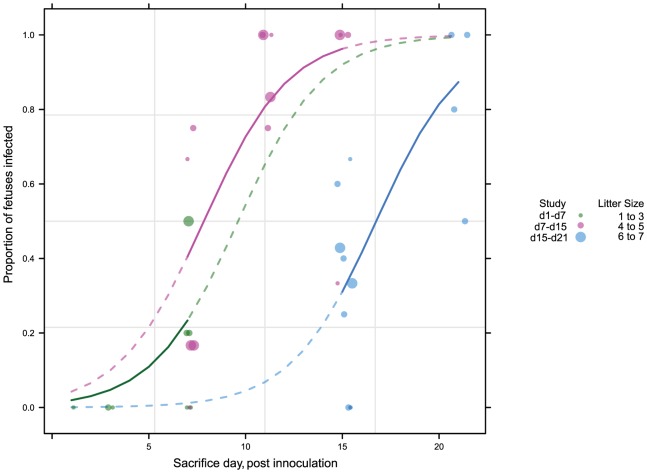
Viral kinetics of fetal infection over 21 days plotted from three separate studies. 43 pregnant guinea pigs were inoculated by subcutaneous injection with 4×10^3^ PFU of pathogenic stock IVP8 at day 21 gestation and sacrificed at 1, 3, 7, 11, 15, and 21 days post-infection. Each glyph (dot) represents the proportion of infected fetuses, with the size of the glyph proportional to the litter size. The solid lines represent the proportion of infected fetuses in a litter from a beta-binomial model fit to all three cohorts. The dashed segment of each line is extrapolated beyond the cohort data and is model based. See statistical section in methods for more details.

To better understand the kinetics of maternal, placental, and fetal infection, we performed a time course study of infection (Figure S1). 43 pregnant guinea pigs were inoculated with 4×10^3^ PFU of GPCMV IVP8 at day 21 gestation and sacrificed at 1, 3, 7, 11, 15, and 21 days post-infection. Guinea pigs were infected at day 21 of gestation to allow for enumeration of viable fetuses by ultrasound (Video S1; Methods for details). Infection of dams and fetal tissues were determined by PCR analysis. Due to the limitations on the number of GPCMV-free, timed-pregnant animals that could be obtained at a given time, this study was performed as three separate studies with overlapping time points. Given that the kinetics of fetal infection (i.e. slopes of the curves) was similar among the three studies (Figure 2), the data from the three studies was combined.

**Figure 3:**
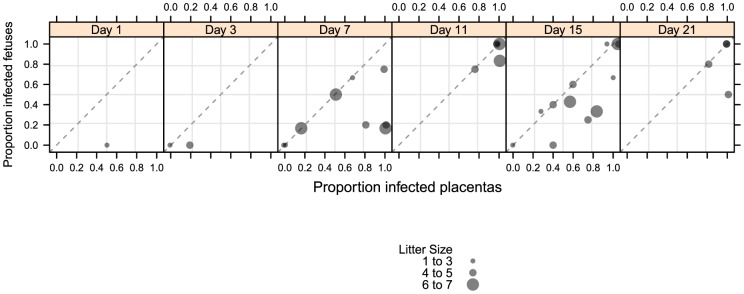
Temporal relationship between placental and fetal infection over 21 days. 43 pregnant guinea pigs were inoculated by subcutaneous injection with 4×10^3^ PFU of pathogenic stock IVP8 at day 21 gestation and sacrificed at 1, 3, 7, 11, 15, and 21 days post-infection. Each glyph represents an infected mother with the size of the glyph proportional to the number of infected placentas and/or fetuses recovered. The proportion of infected placentas (x-axis) and infected fetuses (y-axis) was determined by qPCR and nested PCR, respectively. Data was compiled from three separate experiments.

Dam mortality (1/5) occurred at d11 post-infection, and increased to 38% mortality overall on d21 (Table 1). Neutralizing titer in dams increased over the time course, with minimal titers at days 7 and 11 and robust titer at day 21 (Table 2). Fetal loss was observed as early as d1 (2/19, 10%), and preceded dam mortality, and increased to 74% loss overall by d21 (Table 1). Fetal loss early in the study is likely not attributable to viral infection due to the fact that a similar rate of fetal loss was found in uninfected controls (Table 1). As early as d7, viral genomes (as measured by qPCR) were detected in the maternal salivary glands in 100% (5/5) of the animals, with an average copy number of 1×10^2^ copies/salivary gland. Also at d7, viral genomes were detected in 50% (20/40) of placentas from infected dams which increased to 90% (18/20) by d11 (Table 2). Consistent with the literature [18], viral genome number peaked in maternal blood at d11 with 1×10^4^ copies/mL, followed by a rapid reduction by d15 (data not shown). Peak viral load (d11) was coincident with the first maternal death. Using qPCR, viral genomes were not detectable in the fetus or the amniotic fluid. However, when a more sensitive nested PCR assay was employed, viral genomes could be detected in the fetus as early as d7, with 31% (12/39) of the fetuses infected (Table 2; Figure 3). Moreover, when the nested PCR assay was applied to the maternal salivary glands and the placenta, virus could be detected robustly at d3 post-infection (Table 2; Figure 3). However we can not formally rule out that detection of virus in the placenta early in infection is due to contamination from maternal blood.

**Table 1:** Maternal mortality and fetal loss in GPCMV-infected guinea pigs.

d.p.i.	Dam mortality	Fetal Loss^A^
1	0/5 (0%)	2/19 (10%)
3	0/5 (0%)	2/19 (11%)
7	0/10 (0%)	5/44 (11%)
11	1/5 (20%)	10/26 (38%)
15	2/6 (33%)	13/27 (48%)
21	6/16 (38%)	52/70 (74%)
Mock	0/8 (0%)	4/41 (10%)

A Fetal loss data is only from surviving dams.

**Table 2:** Viral kinetics of GPCMV during congenital infection.

Dams				Fetal Tissue	
d.p.i	SG^A^	NAb^B^	Placenta^C^ ^D^	Fetus^D^	AF^D^ ^E^
1	1/5^D^ (20%)	ND^F^	4/19^D^(21%)	0/17(0%)	ND
3	2/5^D^ (40%)	ND^F^	4/19^D^(21%)	0/17(0%)	ND
7	10/10^C^(100%)	1∶7	20/40^C^(50%)	12/39(31%)	0/39(0%)
11	4/4^C^(100%)	1∶42	18/20^C^(90%)	16/16(100%)	13/16(81%)
15	4/4^C^(100%)	1∶433	9/14^C^(64%)	14/14(100%)	14/14(100%)
21	10/10^C^(100%)	1∶714	17/21^C^(81%)	12/19(63%)	15/18(83%)
Mock	0/8^C^ ^D^(0%)	1∶1	0/37^C^ ^D^(0%)	0/37(0%)	0/37(0%)

A SG, salivary glands from pregnant dams.

B NAb, dam's neutralizing antibody titer, expressed as serum dilution determined by qPCR.

C virus detected by qPCR.

D virus detected by nested PCR.

E AF, amniotic fluid.

F ND, not done.

When the incidence of placental and fetal infection from individual mothers was plotted over time, the following pattern emerges: placental infection occurred without detectable fetal infection (e.g. glyphs plotted below the diagonal line) but fetal infection did not occur without detectable placental infection (e.g. lack of glyphs plotted above the diagonal line) (Figure 3). This pattern suggests a temporal relationship between placental and fetal infection with placental infection occurring prior to fetal infection (Figure 3). In addition, using the nested PCR assay, viral genomes could be detected in the amniotic fluid by day 11, with 81% (13/16) infected (Table 2). Given that the first detectable amniotic fluid infection occurred following fetal infection, these results suggest a sequential order of infection, beginning with maternal salivary gland infection and rapidly spreading to the placenta, the fetus, and then the amniotic fluid.

### Generation of anti-GPCMV monoclonal antibodies

The goal of this study was to evaluate the ability of a neutralizing monoclonal antibody to protect dams and their offspring from infection and loss. GPCMV encodes homologs of the HCMV glycoproteins that mediate viral entry: gB, gH/gL, gO, in addition to UL128, UL130, and UL131A (referred to in GPCMV as GP129, GP131, and GP133) [19]–[25]. In order to develop monoclonal antibodies that neutralize GPCMV, mice were immunized with GPCMV virions or baculovirus-expressed recombinant glycoprotein complexes. The resulting clones were screened for GPCMV neutralizing activity on primary guinea pig fibroblast and endothelial cells. Using this approach, we screened over 5000 hybridoma clones and isolated 6 hybridoma clones that neutralized GPCMV with EC50s (µg/mL) ranging from 0.01 to 2.1 on endothelial cells and 0.07 to 4.1 on fibroblast cells (Table 3).

**Table 3:** Characterization of mouse monoclonal antibodies against GPCMV.

ELISA^A^						FACS^B^	EC50 (µg/mL)^C^	
Clone	Isotype	gB	gHgL	gHgLgO	Pentamer	gHgL	Fibroblast	Endothelial
1282	IgG3, k	−	−	+	−	−	0.82	0.24
1597	IgG2a,k	−	+	+	+	+	1.5	0.12
1968	IgG1, k	−	+	+	+	+	0.65	0.16
1778	IgG, k	−	−	−	−	−	0.33	0.04
1593	IgG2a, k	−	−	−	−	−	0.07	0.01
394^D^	IgG1, k	−	+	+	+	+	4.1	2.1

A ELISA performed with soluble proteins; gB, gH/gL, gH/gL/gO and Pentamer = gH/gL/GP129/GP131/GP133.

B Expression of gH/gL protein on the surface of 293T cells.

C Antibody neutralizing potency as determined by the anti-gB immunofluorescence method.

D Soluble gH/gL protein was used as immunogen.

To determine the antigen specificities of each antibody, binding studies were performed on the following soluble proteins: gB, gH/gL, gH/gL/gO complex, and gH/gL/GP129-133 complex. Binding analysis with soluble glycoprotein complexes revealed that three antibodies recognize the gH/gL heterodimer (1597, 1968, 394) and one clone (1282) recognizes gH/gL/gO but not gH/gL alone, suggesting that the antibody specifically recognizes gO. The remaining two antibodies (1778, 1593) did not react with any of these complexes (Table 3). Further confirmation of these results with antibodies 1597, 1968, and 394 was obtained using FACS analysis on cell surface-expressed gH/gL complex (Table 3). Competition FACS experiments with the anti-gH/gL monoclonal antibodies revealed that 1597 and 1968 compete with each other (data not shown). Extensive characterization of the most potent antibodies, 1778 and 1593, did not reveal their epitopes, and as such, these antibodies were not pursued further. Neutralizing antibodies were not recovered that recognized GPCMV gB or GP129/GP131/GP133. We moved forward with anti-gH/gL clone 1968, due to the fact that it is the most potent antibody with known target specificity.

### Chimerization and characterization of the anti-GPCMV gH/gL monoclonal antibody

**Figure 4:**
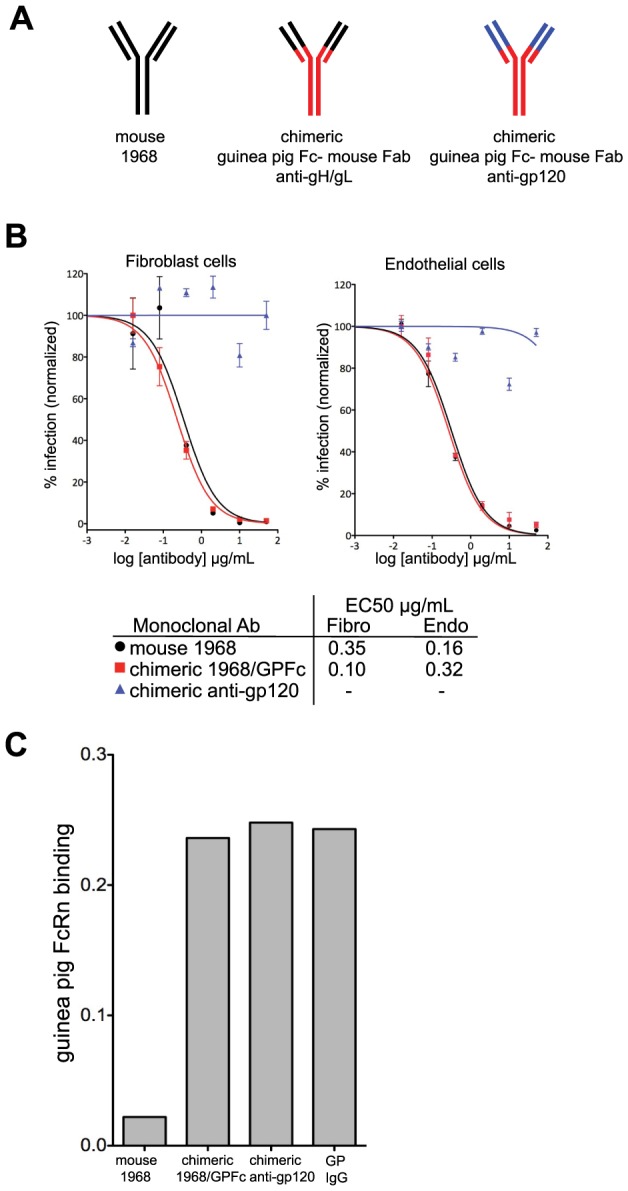
Chimerization of the mouse 1968 monoclonal antibody. (A) Schematic of mouse 1968, chimeric guinea pig-mouse 1968/GPFc, and chimeric guinea pig-mouse anti-gp120 negative control antibodies. The chimeric antibodies are comprised of a guinea pig Fc and a partial variable region from guinea pig IgG2 (red) and the mouse 1968 partial Fab, containing the antigen-binding site (black) or the anti-gp120 partial Fab, containing the antigen-binding site (blue). (B) Neutralization profiles of mouse 1968, chimeric 1968/GPFc, and chimeric anti-gp120 antibodies on primary guinea pig fibroblasts and endothelial cells. Data from two independent experiments are graphed using a non-linear regression analysis for calculating EC50 values. Concentration of monoclonal antibody is provided in µg/ml. (C) Mouse 1968, chimeric 1968/GPFc, chimeric anti-gp120 antibodies or guinea pig polyclonal IgG were analyzed for their ability to bind to soluble guinea pig FcRn/β2-microglobulin complex by Octet Red QK Instrument.

In order for a monoclonal antibody to protect the fetus from infection and/or disease, it needs to be present in the maternal serum. However, a mouse IgG may have poor pharmacokinetics (PK) in the guinea pig for two reasons: 1) mouse IgG does not bind significantly to human FcRn, and by extension may not bind to guinea pig FcRn [26], and 2) the guinea pig may mount an immune response against the foreign mouse antibody, thus rapidly clearing it from circulation. To improve PK, chimeras between the mouse F(ab′)2 and the guinea pig IgG2 constant region were generated (Figure 4A). An irrelevant isotype control directed against HIV gp120 antigen was constructed in parallel (Figure 4A). In a neutralization assay on fibroblast and endothelial cells, the 1968 mouse-guinea pig chimeric antibody (henceforth referred to as 1968/GPFc) retained anti-GPCMV potency similar to the fully-murine 1968 antibody (Figure 4B). 1968/GPFc was also able to neutralize the pathogenic GPCMV stock (IVP8) on fibroblasts with potency similar to the tissue culture-adapted GPCMV strain (strain 22122; Figure S2).

To evaluate the ability of the 1968/GPFc antibody to bind guinea pig FcRn, binding assays were performed. To this end, the guinea pig genomic sequences encoding for the extracellular domain of FcRn and β2-microglobulin were cloned and transiently co-expressed in HEK293 cells (Figure S3). Both the 1968/GPFc and the anti-gp120 chimera were evaluated for their ability to bind the FcRn-β2-microglobulin soluble complex in biosensor assays. Both antibodies bound to FcRn in equal affinity to guinea pig purified IgG from serum, whereas the mouse antibody 1968 failed to bind (Figure 4C).

PK of the 1968/GPFc antibody in guinea pigs was evaluated following a single antibody dose at 10 mg/kg in infected and uninfected pregnant dams (Figure S4). The 1968/GPFc antibody was administered at 1-day post-infection. Blood samples were drawn from each guinea pig before the start of the study and at 0.5 h, 8 h, d1, d7, d14, and d21 post-infection. The 1968/GPFc antibody was detectable in the blood with a half-life of approximately 8.46 days in uninfected guinea pigs (Figure S4). This half-life is considered within normal range for an IgG in guinea pig [27]. Neutralization potency was similar for infected and uninfected animals at day 1 (uninfected, 1∶477; infected, 1∶355) and day 3 uninfected 1∶841; infected 1∶622). We observed an increase of anti-gH/gL antibody concentration after day 15 post-antibody administration only in infected animals. But given that this increase only occurred in infected animals, it is likely due to the presence of maternal anti-gH/gL antibodies in response to the infection. This rise in endogenous anti-gH/gL antibody titer is consistent with our observations from the seroconversion study (Figure 1B).

### A neutralizing monoclonal antibody reduces fetal infection and loss

**Figure 5:**
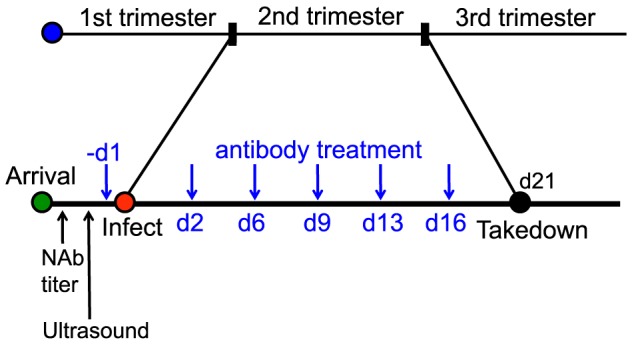
Schematic for the anti-gH/gL prophylactic protection study. 15 GPCMV-free, timed pregnant guinea pigs were inoculated by subcutaneous injection with 4×10^3^ PFU of IVP8 stock at 21 days gestation. 1968/GPFc and control anti-gp120 antibodies were administered I.P. to 7 and 8 guinea pigs, respectively. The first dose was given one day prior to infection and then twice per week for 3 weeks at 8 mg/kg dose for a total of 6 doses. At day 21 post-infection, guinea pigs were sacrificed and viral infection was determined by PCR from the placenta, fetus, and maternal salivary glands.

Given that the 1968/GPFc antibody is potently neutralizing against GPCMV and can bind to guinea pig FcRn, its ability to protect against maternal and fetal death as well as fetal infection was evaluated. To this end, 7 pregnant dams received the 1968/GPFc antibody at 8 mg/kg starting at one day prior to viralinoculation, followed by twice per week injections for a total of 6 doses. The 1968/GPFc antibody was administered as a prophylactic, due to the narrow therapeutic window revealed by the kinetics study. This dose of antibody was estimated to provide a serum concentration >10X in vitro neutralization EC90 at Ctrough (i.e. trough plasma concentration measured at the end of a dosing interval at steady state). In parallel, 8 pregnant dams were administered the anti-gp120 chimera at the same dose for comparison. All 15 pregnant guinea pigs were inoculated with 4×10^3^ PFU of GPCMV IVP8 at day 21 of gestation and sacrificed at day 21 post-infection (Figure 5). Between days 13 and 14, there was mortality in half of the pregnant dams (4/8) in the anti-gp120 group (Table 4). A higher percentage of maternal loss was observed in this study than from in-house historical studies in which the average maternal loss at day 14 was 32% (10/31 dams). In contrast, only 1 out of 7 guinea pigs in the 1968/GPFc group died (Table 4). Although these findings did not reach statistical significance, the reduced death in the 1968/GPFc group is suggestive of dam protection. Of the surviving dams at day 21 post-infection, the average neutralizing viral titer was similar in both groups (1∶840 for 1968/GPFc-dosed animals and 1∶540 for anti-gp120-dosed animals).

**Table 4:** Effect of 1968/GPFc antibody on maternal mortality and congenital GPCMV infection^A^ ^B^.

Treatment group	Dam mortality	Fetal mortality	Placenta^C^	Fetus^D^
1968/GPFc^E^	1/7(14%)	24/37(65%)	13/13(100%)	2/13(15%)
Anti-gp120^E^	4/8(50%)	20/20(100%)	n/a^F^	n/a^F^
Historical studies^G^				43/60(72%)

A Pregnant guinea pigs were infected with 4×10^3^ PFU of IVP8 at the start of the 2^nd^ trimester.

B Cumulative results at 21 days post-infection.

C Virus detected by quantitative PCR.

D Virus detected by nested PCR.

E Antibody administered I.P. one day prior to infection at 8 mg/kg dose and then twice per week for 3 weeks with a total of six doses (see Figure 5 for study scheme).

F n/a, not applicable, no fetuses were alive at end of study.

G Averaged in-house historical data from infected pregnant guinea pigs without antibody treatment.

In addition to mother mortality, fetal loss and infection were also measured. 100% fetal loss was observed in the anti-gp120 group, whereas only 65% of the fetuses were lost at the time of sacrifice in the 1968/GPFc group (Table 4). Since all of the fetuses were lost in the anti-gp120 control group, the infection rate of fetuses from the 1968/GPFc group was compared to those from in-house historical controls. Two in-house studies of congenital infection were averaged to provide a historical reference (Table 4). The 1968/GPFc group showed a significant reduction in fetal infection, with 15% of the fetuses infected as compared with 60% of historical controls (p = 0.03) (Table 4). Although protection was observed, it is noted that the fetuses from the 1968/GPFc group were recovered from two dams, providing a limited data set from which to derive a p-value. The 1968/GPFc antibody did not appear to block viral placental entry, with 100% of the placentas infected in the 1968/GPFc group. These results suggest that the presence of 1968/GPFc neutralizing antibodies reduce the rate of GPCMV fetal infection and loss, and in addition, may improve dam survival.

## Discussion

A therapeutic for HCMV disease is a major public health priority given the disability in newborn infants caused by congenital infection. Both in humans and in guinea pigs, passive administration of anti-CMV polyclonal antibodies has been shown to be protective to the fetus [1], [11]. A neutralizing monoclonal antibody has not been evaluated in clinical trials for this indication nor has been tested in an animal model of congenital infection. In this study, we evaluated the ability of an anti-gH/gL neutralizing monoclonal antibody to protect against fetal loss and infection in the guinea pig model of congenital CMV. Several guinea pig studies have delineated a role for antibodies in protecting the fetus from GPCMV infection and disease [1]–[3]. In early studies, Bia et al. demonstrated that preconception infection protected against in utero GPCMV transmission, most likely due to the generation of neutralizing antibodies in the mother [17], [28]. More recently, studies determined that passive immunization of polyclonal anti-GPCMV antibodies (or anti-gB polyclonal antibodies) were protective in the congenital setting [1], [3]. Our studies build on these pioneering studies and demonstrate that a neutralizing monoclonal antibody can show efficacy in the congenital setting. These studies support the hypothesis that a neutralizing antibody response that targets a single epitope can be protective against fetal infection. By extension, a neutralizing monoclonal may be efficacious in the congenital setting in humans as well.

In this study, we delineated the kinetics of seroconversion andcongenital infection. We found that seroconversion and maternal-fetal transmission of GPCMV occurred at approximately in the same time frame (<7 days). Our results are consistent with the time course of transmission established by Griffiths et al., (1980), with the peak of fetal infection occurring at 11–15 days post-infection [29]. However, our study increases the understanding of the kinetics of primary infection via greater resolution of time points and the utilization of molecular techniques. The rapid viral spread to the placenta necessitated a prophylactic rather than therapeutic study. Altering the infection route or significantly reducing the inoculum titer to reduce the rate of viral spread unfortunately did not result in robust fetal infection (data not shown). In fact, viral kinetics may in part explain the marked reduction of protection observed by Bratcher et al., (1995), when directly comparing the therapeutic versus prophylactic administration of hyperimmuneglobulin in guinea pigs [1].

We isolated and characterized six potently neutralizing monoclonal antibodies against GPCMV: three against the gH/gL protein, one against gO protein, and two with unknown targets. One of the anti-gH/gL antibodies, 394, was isolated from mice immunized with gH/gL protein rather than whole virus. It is interesting to note that antibody 394 has significantly less neutralization potency than the other anti-gH/gL antibodies resulting from whole virus immunization. This suggests that viral soluble complexes may not serve as the most effective immunogens for the generation of neutralizing antibodies. Surprisingly, we did not recover any neutralizing antibodies against gB or the GP129/GP131/GP133 proteins. Antibodies against the HCMV homologs of these proteins have been shown to be highly neutralizing [30], [31]. However, one formal possibility is that our most potent antibodies (1778, 1593) may recognize epitopes on gB or the GP129/GP131/GP133 complex that are not identified by soluble or cell-associated complexes. Our inability to recover neutralizing antibodies against this complex may reflect that GPCMV gH/gL/UL129/UL131/UL133 complex is not essential for viral entry in fibroblast and epithelial cells (we conducted our screens on these cell types) [23].

Here, we have shown that a highly neutralizing monoclonal antibody against GPCMV gH/gL reduces fetal infection rate and death following maternal GPCMV challenge. Despite the protection observed, we were surprised by the inability of the anti-gH/gL monoclonal antibody, 1968/GPFc, to protect against placental infection. In humans, infection of the fetus most likely occurs by transcytosis across the placenta, in which the virus takes advantage of low avidity antibodies for transport [32]. High avidity, neutralizing antibodies have been shown to intercept this pathway, and ultimately may prevent fetal infection [32]. Mothers with preconception immunity to HCMV have babies with a much lower incidence of infection and disease, suggesting that maternal antibodies can be protective in this setting. In contrast, placental infection in the guinea pig is rapid and direct following subcutaneous inoculation. In a non-laboratory setting, spread between animals may result from a lower titer of inoculum via mucosal membranes and may ultimately lead to a slower progression of the infection, possibly allowing better protection by antibodies. A second possible explanation is that full protection may require administration of an antibody cocktail. Consistent with this possibility, Chatterjee et al, found that administration of high titer anti-gB polyclonal serum, which presumably binds multiple neutralizing epitopes, significantly reduced placental infection and prevented fetal infection [3]. However, there are limitations in drawing such comparisons due to differences in study methodology.

In humans, humoral responses to natural infection set the bar for vaccines or immunotherapeutics as natural immunity is known to prevent and reduce disease. Along these lines, we found that the serum neutralizing activity of guinea pigs administered with 1968/GPFc on d21 of gestation was approximately equal to that of infected animals treated with control antibody on d21 post-infection (i.e. d42 of gestation). In addition, we found that this serum neutralizing activity was similar to that from GPCMV positive guinea pigs procured from our vendor (data not shown). Indeed, the concentration of anti-gH/gL antibody in guinea pigs administered with 1968/GPFc is similar to infected control guinea pigs on d21 (200 ug/ml for guinea pigs administered 1968/GPFc vs 120 ug/ml for guinea pigs administered control antibody). Despite these similarities at d21, the key difference between animals developing their own immunity to GPCMV versus the administration of 1968/GPFc is *timing*: animals administered 1968/GPFc have a neutralizing serum titer significantly higher than that of naïve animals from the onset of infection, thereby providing immediate protection to the developing fetus. Along these lines, guinea pigs that are seropositive prior to conception, and thus have neutralizing titers throughout gestation, also result in protection of the fetus [29]. Given that passive antibody administration of an anti-gH/gL monoclonal antibody can provide levels of neutralizing titers similar to that of seropositive animals, these results may have important implications therapeutic development.

An anti-HCMV neutralizing monoclonal antibody has not been evaluated in clinical trials for the prevention of congenital HCMV. However, MSL-109, a neutralizing monoclonal against HCMV gH, has been evaluated in the prevention of HCMV infection following allogenic hematopoietic stem cell transplantation and as adjuvant therapy for HCMV retinitis in HIV-infected individuals without apparent benefit [33], [34]. Recently, a nongenetic mechanism of generation of viral resistance was demonstrated in vitroand was proposed to explain the apparent clinical failure of MSL-109 [35]. However, analysis of a subset of the transplantation patients who were at high risk for primary HCMV infection (donor positive/recipient negative patients, D+/R−) actually demonstrated that MSL-109 could confer protection [33]. Since HCMV congenital infection can also result from primary HCMV infection in the mother, this suggests that monoclonal antibody therapy might be useful in this setting. Given that our study did not directly compare antibodies neutralizing different glycoprotein epitopes, we are not claiming that an anti-gH/gL antibody is the optimal therapy for congenital HCMV. However, a recent vaccine study strongly suggests that the pentameric gH complex is the primary target for neutralizing antibodies [36]. Our study suggests that a therapeutic that targets a single neutralizing epitope on HCMV (or alternatively, a cocktail of monoclonal antibodies) may have clinical benefit. Obviously, it will take a human trial to determine if a monoclonal antibody is indeed protective, but the results from this study are consistent with this hypothesis.

## Materials and Methods

### Animals

All animal work has been conducted on an approved protocol, reviewed and approved by Genentech's Institutional Animal Care and Use Committee (IACUC studies: 11-2904, 10-1827, 10-1006A, 10-0499A). Genentech, Inc. is registered with the USDA and its protocols adhere to the USDA regulation of the Animal Welfare Act and Animal Welfare Regulations. Genentech, Inc. is OLAW (Office of Laboratory Animal Welfare) assured and protocols adhere to the Public Health Service (PHS) Policy on the Humane Care and Use of Laboratory Animals. Timed-pregnant Hartley guinea pigs were obtained at 15 days of gestation from Elm Hill Breeding labs (Tynsboro and Chelmsford facilities in MA) and housed under conditions approved by the American Association of Accreditations of Laboratory Animal Care Committee. As guinea pigs do not have a genital plug, Elm Hill Labs determines the gestation stage by the timing of the previous birth: guinea pigs typically breed 24 hours post-birth if a male is placed in the cage with the female. In addition, Elm Hill labs palpates the females to confirm pregnancy. Prior to their use, all animals were determined to be GPCMV-free by neutralization assay. In addition, we confirmed pregnancy in-house via ultrasound by which we only used guinea pigs that were: 1) pregnant, 2) had fetuses of a similar stage and size (this can be measured using ultrasound), and 3) had viable fetuses. Of note, most pregnant females had fetuses of similar size appropriate of the expected gestational stage. Ultrasound imaging was performed using an Acuson Sequoia C512 ultrasound imaging system with a 15L8-S probe (Siemens Medical Solutions, Malvern, PA, USA) or Vevo2100 imaging system with a 33 MHz probe (Visualsonics, Toronto, CN). Male Hartley guinea pigs obtained from the same facilities were used for in vivo passaging of the virus.

### Virus and cells

HEK293T cells were obtained from ATCC (CRL-11268) and cultured in DMEM+10% FBS. CHO cells were obtained from ATCC and cultured in F-12K media+10% FBS. Guinea pig embryonic fibroblasts (gefs) and primary endothelial cells were generated in-house according to Auerbach et al. (2013) [23] GPCMV (strain 22122, American Type Culture Collection) was propagated on gefs. Viral stocks were titered at 24 hours post-infection (hpi) using immunofluorescence microscopy with an anti-GPCMV gB monoclonal antibody (29-29; gift of Bill Britt, University of Alabama) [23], [24]. Viral stocks had infectivity titers of 10^5^–10^6^ PFU/ml on fibroblast and endothelial cells. This viral stock was used to prepare the salivary gland-derived GPCMV stock of higher virulence by 8 sequential passages *in vivo* in Hartley male guinea pigs as previously described [37]. We refer to this pathogenic stock as IVP8. Briefly, guinea pigs were infected subcutaneously with approximately 10^5^ PFU. After 21 days of infection, the salivary gland was removed, homogenized, sonicated, clarified, titered and used for re-infection. After 8 sequential passages, salivary gland extract was stored in frozen aliquots at −80°C at 1∶1 in 0.2M sucrose phosphate buffer at 10^5^ PFU/ml. A single pool of extract was used throughout this study.

### Seroconversion analysis

15 pregnant guinea pigs were inoculated by subcutaneous injection of 4×10^3^ PFU of IVP8 stock at 21 days gestation. Blood samples (approximately 400 µl each) were collected for ELISA analysis via the orbital route before the start of the study (pre-immune negative control samples) and at d3, d7, d10, d14, d17, and d21 post-infection. 15 pregnant guinea pigs were divided into two groups to alternate bleeding: Group 1 (n = 7) was bled on d3, d10, d17, and Group 2 (n = 8) on d7, d14. Guinea pigs from both groups were bled and sacrificed at d21 post-infection. Four pregnant guinea pigs died before the end of the study between d12-17 resulting in 27% dam mortality (similar mortality to our in-house historical studies). The pre-immune uninfected pregnant guinea pig serum was used for negative controls to determine background levels. Serum samples were diluted in PBS+0.5% BSA+0.25% CHAPS+5 mM EDTA+0.35M NaCl+10 ppm Proclin+0.05% Tween 20 at pH 7.4 and analyzed by ELISA using soluble GPCMV gB or gH/gL protein (see ELISA section for details). Serum interference was evaluated and donkey anti-guinea pig IgG (H&L) provided the best signal. Samples were diluted starting at 1/100 and then serially diluted 3-fold for a total of 8 points. The minimum dilution was found to be 1/2700 for the anti-gB IgG response and 1/100 for the anti-gH/gL IgG response. Relative OD readings were plotted in lieu of absolute antibody concentrations because of the challenges in obtaining polyclonal anti-gB and anti-gH/gL purified standards. Pre-immune samples (e.g. serum from uninfected pregnant guinea pigs) and day 12 positive serum were included as controls in each assay. This allowed for normalization across all ELISA plates.

### GPCMV kinetic analysis in pregnant animals

43 pregnant guinea pigs were inoculated by subcutaneous injection with 4×10^3^ PFU of pathogenic stock IVP8 at day 21 gestation and sacrificed at 1, 3, 7, 11, 15, and 21 days post-infection. Guinea pigs were infected at day 21 of gestation to allow for enumeration of viable fetuses by ultrasound. Infection of dams was verified post-sacrifice by determination of viral copy number from maternal salivary gland homogenates. Due to the limitations on the number of GPCMV-free, timed-pregnant animals that could be obtained at a given time, this study was performed as three separate studies with overlapping time points. Despite experiment-to-experiment variation in mother survival and fetal loss in groups sacrificed on different days, the kinetics of fetal infection (i.e. slope of the curves) was similar among the three experiments, allowing for studies to be combined (Figure 2). In parallel, 8 dams were mock infected (with DMEM media) and sacrificed at 21 days post-infection.

### Neutralization assays

All anti-GPCMV antibodies from hybridoma screens were evaluated by neutralization assays based on those with HCMV as described by Abai et al. (2007). Briefly, antibody was serially diluted in complete media and mixed with virus diluted in complete media such that the final virus concentration resulted in approximately one infectious virus per cell (Multiplicity of Infection (MOI) = 1) when mixed with media. Antibody and virus were mixed and incubated at 37°C for 1 hour prior to incubation for 24 hours on a confluent monolayer of cells. 24 hours post-infection, cells were fixed with 100% ethanol and blocked in PBS and 2% bovine serum albumin (BSA) and then stained with monoclonal anti-guinea pig gB antibody (29-29; gift of Bill Britt, University of Alabama) [24]. Cells were washed with PBS and incubated with the appropriate AlexaFluor 488 and Hoechst (Invitrogen) stains. Cells were imaged and counted using the ImageXpress Micro and MetaXpress software (Molecular Devices; Sunnyvale, CA).

All guinea pig sera (e.g. serostatus screening; kinetic analysis of infection; protection study) and tissue homogenate (e.g. neutralization of IVP8 by 1968/GPFc) were evaluated for presence of neutralizing antibodies by an in-well-lysis-TaqMan qPCR assay. In-well-lysis-TaqMan qPCR assay was used as an alternate to the immunofluorescence assay due to the high background when working with serum and tissue. Serum was serially-diluted in 5-fold dilutions in DMEM media, and mixed with virus (MOI = 1). The mixture was incubated for an hour at 37°C prior to incubation for 48 hours on a confluent monolayer of guinea pig primary cells. Cells were lysed using the Cells-to-CT kit (Ambion, Austin, TX) and the relative percentage of infection was quantified in reference to negative controls (e.g., no serum, uninfected guinea pig serum). A multiplex qPCR using TaqMan probes (Applied Biosystems, Carlsbad, CA) targeting the GPCMV gene *GP83* and the endogenous control, guinea pig β-actin, allowed for analysis using the comparative ΔΔCT method [38]. qPCR reactions were performed in an ABI 7500 real-time PCR system and then analyzed using the ABI analysis software (Applied Biosystems). See the qPCR assay section below for primer sequence and PCR conditions. All assays were carried out in duplicate, and the results are expressed as the normalized percent of infection. Neutralization data was analyzed with Prism 5.0 (GraphPad Software; La Jolla, CA) using non-linear regression (curve fit; 4-parameter).

### Monoclonal antibody generation

Ten Balb/c mice (Charles River Laboratories International, Inc., MA, USA) were immunized intraperitoneally with 10^6^ PFU/mice/injection of GPCMV whole virus (strain 22122) in an adjuvant containing metabolizable squalene (4% v/v), Tween 80 (0.2% v/v), trehalose 6,6-dimycolate (0.05% w/v) and monophosphoryl lipid A (0.05% w/v; all components obtained from Sigma Aldrich, USA). In addition, in order to generate antibodies against the gH/gL and gH/gL/GP129/GP131/GP133 complexes, we immunized a subset of mice with soluble gH/gL or gH/gL/GP129/GP131/GP133 proteins at 2 µg of protein/mouse using the same adjuvant that was used for the whole virus immunizations [23]. The mice were boosted with the virus or soluble protein, with adjuvant, twice per week. Following 10 injections, serum samples were evaluated for viral neutralizing activity *in vitro* using primary guinea pig endothelial cells. B cells from spleens harvested from three mice demonstrating neutralizing serum activity were then fused with mouse myeloma cells (X63.Ag8.653; American Type Culture Collection, Manassas, VA, USA) by electrofusion (Hybrimmune, ECM 2001; Harvard Apparatus, Inc., Holliston, MA, USA). After 10–14 days the supernatants were harvested and screened for IgG production by a direct ELISA. All ELISA positives (i.e. cells producing IgG) were re-screened to evaluate viral neutralizing activity. Clones demonstrating the desired neutralizing activity were then subcloned by limiting dilution (single cell/well) and retested as described above. The final clones were cultured in INTEGRA CELLine 1000 bioreactors (INTEGRA Biosciences AG, Zizers, Switzerland). The supernatants were then purified by affinity chromatography (MabSelect SuRe; GE Healthcare, Piscataway, NJ, USA), sterile-filtered (0.2 µm), and stored at 4°C in PBS. All monoclonal antibodies were characterized by ELISA and FACS (see Methods sections below).

### Baculovirus production of GPCMV glycoproteins

The production and characterization of recombinant GPCMV gB, gH, gL, GP129, GP131, and GP133 proteins have been described in Auerbach et al. (2013) [23]. Briefly, each gene was cloned into pAcGP67 for expression in the baculovirus system with a C-terminal His tag. Native signal sequences were removed and replaced with the insect signal sequence. gB, and gH transmembrane domains were eliminated to maximize secretion into the media. Plasmids were transfected into *Spodoptera frugiperda* (Sf9) and *Trichloplusia ni* (Tni) cells (Expression Systems LLC), passaged 3 times and stored in 2% HIFBS. For protein expression, cells were infected, incubated at 37°C and supernatant was harvested at 72 hrs post infection. Protein was then purified over nickel resin and analyzed by SDS-PAGE and Western blot.

### ELISA using soluble GPCMV glycoproteins

96- or 384-well Nunc Maxisorp plates were coated with the following baculovirus-generated proteins: gB at 2 µg/ml, gH/gL at 0.5 µg/mL, gH/gL/gO at 0.1 µg/mL, or gH/gL/GP129/GP131/GP133 0.1 µg/mL in 0.05M Sodium Carbonate Buffer, pH 9.6. Plates were washed three times with PBS/0.05% Tween 20 and blocked with 50 µL of PBS/0.5%BSA/10 ppm Proclin. Guinea pig serum samples or anti-GPCMV monoclonal antibodies were allowed to bind to viral antigens for two hours and the plates were rinsed six times with wash buffer. Goat anti-guinea pig IgG (H&L) conjugated to horseradish peroxidase (HRP) (Jackson ImmunoResearch) at 40 ng/ml was added for one hour to detect the GPCMV-specific IgG antibodies. The plates were washed 6 times prior to addition of and TMB substrate (Moss Inc; Pasadena, MD). The reaction was stopped after 10 minutes with equal volume 1M phosphoric acid and absorbance was read at 450 nm and referenced at 620 nm.

### Expression and detection of GPCMV glycoproteins at the cell surface

Plasmids containing gH+gL glycoproteins from GPCMV were constructed such that each protein was expressed in equal stoichiometry by separating each gene with a “self-cleaving 2A peptide” [39]. The plasmid contains GPCMV gH+gL+eGFP (cloned from cDNA). Plasmid was transfected into human embryonic kidney (HEK)-293T cells (ATCC) using Lipofectamine 2000 (Invitrogen; Carlsbad, CA) to express GPCMV gH/gL complex at the surface. After 2 days, cells were dissociated and stained. Fluorescence of individual cells was measured using FACSCalibur (BD Biosciences) and analyzed using FlowJo software (Tree Star).

### Chimerization of mouse anti-gH/gL monoclonal antibody

At the time of this work, only the protein sequences of the guinea pig IgG Fc regions were published. The protein sequences of the constant region of guinea pig IgG1, human IgG1, murine IgG2a and rabbit IgG1 were aligned and used to design degenerate PCR primers to regions of homology. Once a fragment of the constant region was cloned, 5′ and 3′ RACE PCR was used to determine the nucleotide sequence of the entire constant region. Two guinea pig antibody heavy chain isotypes (IgG1 and IgG2) were identified. Due to its sequence similarity to human IgG1, guinea pig IgG2 was used. The nucleotide sequence for IgG2 was submitted to the GenBank database and assigned accession numbers KF491482 (heavy chain) and KF491483 (light chain).

The variable heavy and light chain domains of hybridoma cell line 1968 were cloned directly from cells using a 5′RACE protocol. The PCR amplified products were subcloned into TOPO-TA (Invitrogen, Invitrogen, Carlsbad, CA) transformed into bacteria and plated on agar. Individual colonies were propagated to obtain plasmid DNA from which the DNA sequences of the subcloned heavy and light chain could be determined. Based on the sequencing information, nested PCR primers were designed to allow restriction digest free cloning of the heavy and light chain domains into mammalian expression vectors encoding the guinea pig IgG2 heavy constant and kappa constant region. The restriction digest free cloning ensured that the entire murine variable heavy and light chain were inserted without sequence modification and seamlessly joined to the guinea pig constant domains. The 1968 mouse-guinea pig chimeric antibody is referred to as 1968/GPFc. A negative control murine-guinea pig IgG2 chimeric plasmid was assembled in a similar fashion. Here, restriction free cloning was initiated from earlier cloned heavy and light chain expression plasmids of a murine antibody directed against gp120 of HIV-1.

### Cloning guinea pig FcRn and β2-microglobulin

The DNA sequence of the guinea pig β2-microglobulin was available from Genbank (accession number AF148875.1). PCR primers were designed for the amplification of guinea pig β2-microglobulin from a commercially available liver cDNA library. The β2-microglobulin gene was amplified by PCR, cloned into a mammalian expression vector, and sequenced. The sequence of the extracellular domain of guinea pig FcRn was identified by PCR with degenerate oligonucleotides designed from regions of homology based on an alignment of rabbit, human, cynomolgus monkey, bovine, sheep, rat and mouse FcRn. The final product from 5′ and 3′ RACE PCR was cloned into a mammalian expression vector and co-transfected into CHO cells with the β2-microglobulin gene (gene sequence obtained from Genbank). Cells were allowed to express the FcRn complex for 5 days. Purified guinea pig FcRn complex was acquired by filtering the cell supernatants, then affinity purifying on human IgG columns at pH 6. Mass spectrometry of a deglycosylated and reduced sample showed the expected molecular weights of the two chains. The nucleotide sequence for guinea pig FcRn has been submitted to the GenBank database and assigned accession number KF491481.

### FcRn complex binding measurements

The binding of antibodies to guinea pig FcRn complex was evaluated using an Octet Red QK instrument: a real-time, label-free platform that evaluates protein-protein interaction. Briefly, the monoclonal antibodies were diluted to 30 µg/mL in 2-(N-morpholino)ethanesulfonic acid (MES, pH 5) buffer and were coupled onto amine reactive biosensors (ForteBio) for 15 minutes. The excess reactive sites on the sensors were blocked for 5 minutes with 1M ethanolamine-HCl (pH 8.5). The coated biosensors were then equilibrated in PBS (pH 6) for 10 minutes so as to wash away excess ethanolamine. Association between the antibodies and FcRn complex was evaluated by dipping the coated biosensors in 30 µg/mL FcRn complex in PBS (pH 6) for 20 minutes. The biosensors were then dipped in PBS (pH 6) for 20 minutes to evaluate dissociation. The FcRn-binding signals were normalized in the following manner: the signals obtained from FcRn-binding to IgG were divided by the signals obtained from total IgG on the biosensor.

### Pharmacokinetics study in pregnant guinea pigs

GPCMV-free, timed pregnant guinea pigs were inoculated by subcutaneous injection with 4×10^3^ PFU of IVP8 stock (4 guinea pigs) or media as a mock-infection control (3 guinea pigs) at 21 days gestation. Guinea pigs were administered with 1968/GPFc antibody at 10 mg/kg at 1-day post-infection. Blood samples (approximately 400 µl each) were collected for ELISA analysis via the orbital route prior to dosing (0 h) and at the following times after dosing with 1968/GPFc antibody: 0.5 hr, 8 hr, d1, d3, d7, d14, and d21. The serum samples were diluted in PBS+0.5%BSA+0.25% CHAPS+5 mM EDTA+0.35M NaCl, +10 ppm Proclin+0.05% Tween 20 at pH 7.4 and analyzed by ELISA using soluble GPCMV gH/gL protein (see ELISA section for details). Serum interference was evaluated with multiple conjugates from Jackson and Novus prior to sample evaluation with the Donkey anti-guinea pig IgG (H&L) HRP providing the best signal. The minimum dilution was found to be 1/400 with a limit of quantification at 0.62 ug/mL. Standard curve range is at 1–100 ng/mL. Samples were diluted starting at 1/400 and then serially diluted 1/3 for a total of 8 points. Dilutions that fell within the standard curve were averaged to give the final concentration. Sera from seronegative animals were used to determine background levels. Serum concentration profiles for each guinea pig were analyzed individually, and mean (s.e.m.) values for the PK parameters were reported.

### Monoclonal antibody protection study

15 GPCMV-free, timed pregnant guinea pigs were inoculated by subcutaneous injection with 4×10^3^ PFU of IVP8 stock at 21 days gestation. 1968/GPFc and control anti-gp120 antibodies were administered I.P. to 7 and 8 guinea pigs, respectively. The first dose was given one day prior to infection and then twice per week for 3 weeks at 8 mg/kg dose for a total of 6 doses. At day 42 of gestation, guinea pigs were euthanized and GPCMV DNA copy number was determined in the maternal salivary glands and placenta by qPCR and fetal tissue and amniotic fluid by nested PCR (see PCR sections below for more details).

### Preparation of DNA from tissue and blood samples

Maternal salivary glands (parotid, submandibular and sublingual glands were combined), placenta, and fetal tissue (the entire fetus except the head) were homogenized with gentleMACS Dissociator (MACS Miltenyi Biotec). Salivary gland homogenates were centrifuged to remove debris. 200 µl of the homogenized tissue or 200 µl of amniotic fluid was used for DNA isolation with Qiagen DNeasy blood and tissue kit. DNA samples were analyzed using real-time quantitative PCR or nested PCR. DNA from whole blood was purified using the QIAamp DNA blood kit.

### Quantitative PCR assay and determination of viral genome copy number

The quantitative PCR (qPCR) assay targeting the GPCMV *GP83* gene for quantification of GPCMV DNA was performed using standard ABI TaqMan protocols. TaqMan primer probe sets were as described by Katano et al. [40]. The primers internal to *GP83* were used for qPCR: GP83F, 5′-CGACGACGACGATGACGAAAAC, and GP83R, 5′-TCCTCGGTCTCAACGAAGGGTC with the addition of the FAM probe 5′-ATCCGAGTTAGGCAGCG. To normalize and/or obtain the GPCMV DNA copy numbers in a single cell, copy numbers of the guinea pig actin gene (GenBank accession number AF508792) were determined by qPCR using primers 5′-TGGATCGGCGGCTCTATC and 5′-CATCGTACTCCTGCTTGCTGAT with the VIC probe 5′-CACTCTCCACCTTCC. As noted above in the Neutralization Assays section, when relative percentages of infection were determined for neutralization assays, multiplex qPCR was performed with both primer/probe sets. In contrast, single primer/probe sets were used when determining absolute copy number (e.g. from guinea pig tissue samples). Both the *GP83* and actin genes were cloned into TOPO vector (Life Technologies) and used for making a standard curves to determine absolute viral copy number.

### Nested PCR assay

Genomic DNA was analyzed from fetal tissue and amniotic fluid using the following sets of primers specific for the GPCMV *GP83* gene. For the first round of PCR the following primers were used: GP83-1, 5′- CCAACGTTCTCGGCCTGACGTTA, and GP83-2, TGGGTACGCCGTCGAACC (targeting a 972 bp product). The qPCR primers described above were used for the second round PCR reaction yielding a 248-bp product. PCR products from the first round were diluted 1∶10 and 5 µl of this was used in the 2^nd^ round of PCR. Reactions and thermocycling conditions followed standard protocol using Phusion high-fidelity DNA polymerase (New England BioLabs) except for annealing temperatures and cycle numbers, which were 65°C and 30 cycles for the first round, and 60°C and 20 cycles for the second round. PCR products were analyzed on 2% agarose gels. Negative and positive controls at both rounds were included.

### Statistical analyses

Two main statistical analyses were done: the first was using the “bridged” kinetics studies (Figures 2 and 3) and the second was to analyze the difference in infection rates with and without a prophylactic at day 21 (i.e. the protection study). Since both analyses were focused on the fetuses, we had to take into account that fetuses are nested within mothers, and as such, should not be counted independently. A common approach for this situation is a beta-binomial model, where within each dam the infections of the fetuses are binomial and the proportion of fetuses infected over all the dams comes from a beta distribution [41]. For the kinetics experiment we fit a model specifying infection rate as a function of takedown time and cohort to account for experimental variation across cohorts. In the protection experiment we used treatment and cohort as terms in the model. Other simple analyses done were to look at mortality in the dams, as that is an indicator of how virulent the virus is within a cohort and abortion rate between infected and unaffected animals. Virulence, as a function of dam mortality, is confounded with cohort, so could not be used to adjust our models further.

## Supporting Information

Figure S1 Schematic for kinetics of GPCMV infection in pregnant guinea pigs. Timed pregnant, GPCMV-free guinea pigs were enrolled at gestation day 21 and were infected subcutaneously with 4×10^3^ PFU of IVP8. Guinea pigs were sacrificed at d1, d3, d7, d11, d15, and d21 post-infection and viral infection was determined by PCR from the placenta, fetus, amniotic fluid, and maternal salivary glands.
(EPS)

Figure S2 Neutralization profiles of the chimeric 1968/GPFc on primary guinea pig fibroblasts on the tissue-culture adapted GPCMV strain 22122 from American Type Culture Collection (red) and IVP8 stock, the pathogenic salivary gland-passaged GPCMV (blue). Data from two independent experiments are graphed using a non-linear regression analysis for calculating EC_50_ values. Concentration of antibody is provided in µg/mL.
(EPS)

Figure S3 Characterization of guinea pig FcRn and beta-2-microglobulin. Protein alignment of human and guinea pig FcRn extracellular domans (A) and β2-microglobulin (B). Residues shaded gray are conserved. (C) FcRn and β2-microglobulin (abbreviated as β2M) co-expressed in CHO cells, affinity purified, and verified by Coomassie-stained protein gel electrophoresis.
(EPS)

Figure S4 Pharmacokinetics (PK) of the 1968/GPFc monoclonal antibody in infected and uninfected pregnant guinea pigs. (A) PK study schematic. GPCMV-free, timed pregnant guinea pigs were inoculated by subcutaneous injection with 4×10^3^ PFU of IVP8 stock (4 guinea pigs) or media as a mock-infection control (3 guinea pigs) at 21 days gestation. Guinea pigs were administered with 1968/GPFc antibody by intraperitoneal injection at 10 mg/kg at 1-day post-infection and bleeds were taken at 0.5 hr, 8 hr, d1, d3, d7, d14, and d21 post-infection. (B) Mean serum concentration-time profiles shown in uninfected (blue) and infected (red) guinea pigs with a single dose of antibody, as measured by ELISA on soluble gH/gL protein.
(EPS)

Video S1 High resolution ultrasound cine clip of guinea pig fetus. B-mode image in gray with power doppler blood signal overlay in red. 33 MHz frequency, 12×12 mm field of view, 25 frames per second.
(MOV)
